# Modified Rhodopsins From *Aureobasidium pullulans* Excel With Very High Proton-Transport Rates

**DOI:** 10.3389/fmolb.2021.750528

**Published:** 2021-11-01

**Authors:** Sabine Panzer, Chong Zhang, Tilen Konte, Celine Bräuer, Anne Diemar, Parathy Yogendran, Jing Yu-Strzelczyk, Georg Nagel, Shiqiang Gao, Ulrich Terpitz

**Affiliations:** ^1^ Department of Biotechnology and Biophysics, Theodor-Boveri-Institute, Julius Maximilian University of Wuerzburg, Wuerzburg, Germany; ^2^ Department of Neurophysiology, Physiological Institute, Julius Maximilian University of Wuerzburg, Wuerzburg, Germany; ^3^ Faculty of Medicine, Institute of Biochemistry, University of Ljubljana, Ljubljana, Slovenia

**Keywords:** *Aureobasidium*, black yeast, photoreceptor, microbial rhodopsins, optogenetics, proton channel, membrane trafficking, fungal rhodopsins

## Abstract

*Aureobasidium pullulans* is a black fungus that can adapt to various stressful conditions like hypersaline, acidic, and alkaline environments. The genome of *A. pullulans* exhibits three genes coding for putative opsins ApOps1, ApOps2, and ApOps3. We heterologously expressed these genes in mammalian cells and *Xenopus* oocytes. Localization in the plasma membrane was greatly improved by introducing additional membrane trafficking signals at the N-terminus and the C-terminus. In patch-clamp and two-electrode-voltage clamp experiments, all three proteins showed proton pump activity with maximal activity in green light. Among them, ApOps2 exhibited the most pronounced proton pump activity with current amplitudes occasionally extending 10 pA/pF at 0 mV. Proton pump activity was further supported in the presence of extracellular weak organic acids. Furthermore, we used site-directed mutagenesis to reshape protein functions and thereby implemented light-gated proton channels. We discuss the difference to other well-known proton pumps and the potential of these rhodopsins for optogenetic applications.

## Introduction

Fungi maintain diverse and complex photosensory systems, perceiving different light wavelengths for adapting to different light conditions (Yu and Fischer, 2019). Gene regulation by light plays an important role in fungal physiology influencing central processes in the lifestyle of a fungus. The only photoreceptors in fungi known to perceive green light are fungal rhodopsins [microbial (type 1) rhodopsins], 7-transmembrane proteins with a retinal moiety covalently bound *via* a protonated Schiff base to a lysine residue in the opsin apo-protein (Ernst et al., 2014). For a long time, the biological function of rhodopsins remained unresolved due to the lack of phenotypes in rhodopsin-KO mutants, but recent investigations suggest a role of rhodopsins in the germination of conidia, plant colonization, and sexual reproduction (García-Martínez et al., 2015; Adam et al., 2018; Brunk et al., 2018; Wang et al., 2018; Panzer et al., 2019).

Mechanistically, fungal rhodopsins are mainly involved in proton translocation (fast photocycle) or sensory perception (slow photocycle) (Fan et al., 2011). Two fungal rhodopsin clades are distinguished, the Nop-1-like [Nop-1 from *Neurospora crassa* (Bieszke et al., 1999)] and CarO-like [related to CarO from *Fusarium fujikuroi* (Prado et al., 2004)] rhodopsins. Within the Nop-1 clade, functionally, NR-like (*Neurospora* rhodopsin) rhodopsins that are slow-cycling sensory proteins and LR-like [related to LR from *Leptosphaeria maculans* (Waschuk et al., 2005)] rhodopsins providing pump functions are distinguished (Adam et al., 2018; Wang et al., 2018). While LR-like rhodopsins show bacteriorodopsin (BR)-like features, proton-pumping CarO-like rhodopsins exhibit a characteristic, as yet only observed among fungal rhodopsins: the pump activity increases in the presence of weak organic acids like acetate, citrate, and the plant hormone auxine indole-3-acetic acid (IAA), suggesting a potential role of rhodopsins during plant infection (Adam et al., 2018). While the rhodopsin clades are clearly separated in ascomycetes, assigning the respective rhodopsins in basidiomyces to these functional groups is more difficult (Panzer et al., 2019).

Microbial rhodopsins play an important role in optogenetics, allowing for the light-controlled manipulation of neuronal functions (Adamantidis et al., 2015). Genes coding for rhodopsins are artificially transferred into the cell, and after heterologous expression, light of different wavelengths is used for modulation of neuronal functions. Using optogenetic functions of defect organs can be partially restored, including vision of blind patients (Sahel et al., 2021). The field of optogenetics is emerging, and with the increasing number of optogenetic applications, the requirements regarding the optogenetic tools are growing as well, especially regarding ion species, wavelength for activation, and pump intensity.

Fungal rhodopsins are only little explored in general, especially in the context of optogenetics. At least one fungal rhodopsin was used in optogenetic applications due to its high pump activity (Chow et al., 2010): LR, also named MAC, and its variants allowed for inhibition of neurons and modulation of *Caenorhabditis elegans* (Chow et al., 2010; Husson et al., 2012). Thus, fungi may provide promising rhodopsin candidates for future applications. Taking the ecological niche of the respective fungus into account can be a further option while looking for good candidates for optogenetic applications.

Certain fungi are assigned to the extremophils, which can survive changing conditions with steep gradients and withstand high salt concentrations and are often exposed to very high radiation (Odoh et al., 2021). Rhodopsins from organisms living under very harsh conditions such as high salt concentrations might behave different from ones colonizing a less challenging habitat.

*Aureobasidium pullulans* is one example of these extremophilic fungi, and advantageously, its genome was recently sequenced and annotated (Gostinčar et al., 2014). The fungus exhibits an enormous stress tolerance and thus also inhabits extreme niches like glacier ice and salines (Zajc et al., 2012). Besides this, *A. pullulans* is frequently used in biotechnological applications for the production of pullulan (poly-⍺-1,6-maltotriose) (Gostinčar et al., 2014). As a producer of melanin, *A. pullulans* also plays a role as an opportunistic human pathogen, especially among immunocompromised patients, where it can provoke not only superficial infections but also disseminated and systemic infections (Chan et al., 2011; Smith and Casadevall, 2019; Wang et al., 2019).

In the genome of the black yeast and ascomycete *A. pullulans*, three putative rhodopsin genes are found, ApOps1 (gb|KEQ89910.1), ApOps2 (gb|KEQ89333.1), and ApOps3 (gb|KEQ87154.1). ApOps1 and 2 are CarO-like rhodopsins, while ApOps3 is assigned to LR-like rhodopsins (Adam et al., 2018).

Searching for rhodopsins in the genomes of extremophilic fungi, we aimed in finding further tools for optogenetic applications and characterized the fungal rhodopsins ApOps1, ApOps2, and ApOps3 of the halotolerant fungus *A. pullulans*. Therefore we improved the membrane trafficking and expressed the cDNA in mammalian cells and *Xenopus* oocytes for electrophysiological analysis. Using patch clamp and a two electrode voltage clamp, we analyzed the pump activity in dependence of wavelength, light intensity, pH, and transported ion species. The ApOpsins were also engineered into proton-specific channels for potential optogenetic manipulation of pH.

## Materials and Methods

### Basic Local Alignment Search Tool, Protein Alignments, and Modeling

We BLAST-searched the genome of *A. pullulans* EXF-150 with the amino acid sequence of different fungal rhodopsins including CarO, LR, and nop-1. The amino acid sequences from BR (gb| AAA72504), CarO (gb| CAD97459), LR (gb| AAG01180), ApOps1 (gb|KEQ89910.1), ApOps2 (gb|KEQ89333.1), and ApOps3 (gb|KEQ87154.1) were aligned using PSI/TM-Coffee alignment (Di Tommaso et al., 2011). ApOps1, ApOps2, and ApOps3 were modeled with the Swissmodel (Biasini et al., 2014) based on the crystal structure of *Coccomyxa subellipsoidea* rhodopsin CsR (6gyh.1.A; ApOps1; Fudim et al., 2019) and LR (7bmh.1.A ApOp2, ApOps3; Zabelskii et al., 2021).

### Molecular Biology

*A. pullulans* EXF-150 cDNA, kindly provided by Cene Gostinčar, was synthesized using SuperScript III first-strand cDNA synthesis kits (Invitrogen) and random hexamer primers (Promega) according to the manufacturer instructions. Coding sequences of ApOps1 and ApOps2 were amplified from cDNA by PCR. ApOps3 was synthesized by ProteoGenix (France). ApOps1 and ApOps2 were cloned into pcDNA5/FRT/TO-CarO::eYFP (García-Martínez et al., 2015) *via BamH*1 restriction sites as described before (Panzer et al., 2019).

The PCR-amplified DNA fragments of ApOps1-3 and CsR [*Coccomyxa subellipsoidea* rhodopsin; the template DNA fragment for CsR was synthesized by GeneArt Strings DNA Fragments (LifeTechnologies, Thermo Fisher Scientific)] were inserted *via Bgl*II and *Xho*I restriction sites into the pGEMHE vectors already containing the cleavable N-terminal signal peptide Lucy-Rho (LR), eYFP, the plasma membrane trafficking signal (T), and the ER export signal (E). Mutations were made by QuikChange site-directed mutagenesis. The generated plasmid sequences were confirmed by DNA sequencing. The linearized plasmids (by *Nhe*I digestion) were used for *in vitro* generation of cRNA with an AmpliCap-MaxT7 high-yield message maker kit (Epicentre Biotechnologies). Afterward, the DNA fragments coding for proton pumps or channels together with fused signal peptides and eYFP were transferred to plasmid pcDNA3.1 (-) *via BamH*I and *Hind*III restriction sites for mammalian cell expression.

For the construction of the fungal-rhodopsin-channelrhodopsin-tandem plasmids, the *Sma*I restriction site in the backbone of pcDNA3.1(-)-hChR2(H134R)-mKate-hbetabR (BL191) (Kleinlogel et al., 2011) was removed by site-directed mutagenesis. In-frame replacement of BR with ApOps1 was enabled by means of overlap extension fusion PCR, yielding pcDNA3.1(-)-hChR2(H134R)-mKate-hbeta-ApOps1. The latter plasmid was used as a template in classical cloning with *Sma*I/*Hind*III restriction sites yielding pcDNA3.1(-)-hChR2(H134R)-mKate-hbeta-CarO, pcDNA3.1(-)-hChR2(H134R)-mKate-hbeta-LR, pcDNA3.1(-)-hChR2(H134R)-mKate-hbeta-ApOps1, and pcDNA3.1(-)-hChR2(H134R)-mKate-hbeta-ApOps2. Fungal LR was amplified from plasmid FCK-Mac-GFP, which was a gift from Edward Boyden (Addgene plasmid # 22223) (Chow et al., 2010).

### Mammalian Cell Culture and Transfection

Initial experiments of nonmodified ApOpsins without 2.0 cassette were performed with human embryonic kidney (HEK) Flp-InTM T-REx™-293 cells (Thermo Fisher Scientific) that were stably transfected with pcDNA™^5^/FRT/TO-ApOps1-eyfp or pcDNA™^5^/FRT/TO-ApOps2-eyfp to obtain the respective HEK293 cell lines following the manufacturer’s instructions. pcDNA3.1(-)-ApOps1-3 2.0 constructs (and for comparison pcDNA™^5^/FRT/TO ApOps1-eyfp and pcDNA™^5^/FRT/TO ApOps2-eyfp) were transiently transfected in NG108-15 cells using Lipofectamine 2000 as described recently (Feldbauer et al., 2016).

### Confocal Laser Scanning Microscopy

Staining procedures and imaging of HEK293 and NG108-15 cells were performed in poly-D-lysine covered in eight-well tissue culture chambers (eight-well tissue culture chamber, Sarstedt, or eight-well Lab-Tek^®^II Chambered # 1.5 German Coverglass System, Nunc™, Thermo Fisher Scientific). NG108-15 cells were seeded in a density of about 10^4^ cells per well and grown for 12–24 h. A confocal laser scanning microscope (SP700, Zeiss, Germany) equipped with three laser lines (488 nm: 10 mW, 555 nm: 10 mW, 639 nm: 5 mW) was used. Images were recorded using a plan-apochromat 63x/1.40 oil M27 objective. The frame size for imaging was set to 1024 × 1024 pixels except otherwise specified, with a bit depth of 16 bits. The PMT detector gain for all channels was 500–700, and laser powers between 1 and 2.8% and pixel dwell times from 1.27 to 3.15 μs were used. Every line was averaged from two recordings with the laser scanning unidirectionally. The pinhole was adjusted to 1 airy unit (AU). Images were processed with ZEN software (ZEN 2012, Zeiss) or Fiji, Version ImageJ 1.50f (Schindelin et al., 2012).

### Oocyte Imaging

Two days after cRNA injection, the expression level of optogenetic tools was evaluated by the fluorescence from the fused YFP. The images of *Xenopus* oocytes were obtained using a Leica DMi8 inverted microscope. An HC FL PLAN 2.5 × 0.07 DRY objective and a DFC3000G camera were used to capture images. The frame size was set to 1296 × 966 pixels, and the digitization was 8 bits. The wavelength of excitation light was 490–510 nm, and the emission channel used was 520–550 nm. The imaging process was performed with Leica Application Suite X software.

### Two-Electrode Voltage Clamp Experiments

cRNA-injected oocytes were incubated in ND96 solution (in mM, 96 NaCl, 5 KCl, 1 MgCl_2_, 1 CaCl_2_, 5 HEPES, pH 7.4) containing 10 µM all-*trans*-retinal at 16°C. Thirty nanograms of cRNA were injected into the *Xenopus* oocyte for all the constructs. Photocurrents were measured 2 days after injection. Illuminations were performed through a 532 nm solid-state laser (Changchun New Industries, China). Electrophysiological measurements with *Xenopus* oocytes were performed at room temperature (20–23°C) with a two-electrode-voltage clamp amplifier (TURBO TEC-03X, npi electronic GmbH, Tamm, Germany). The bath solutions for electrophysiological recording are indicated in each figure legend. All buffers contained 1 mM MgCl_2_, 2 mM CaCl_2_, and 5 mM HEPES. Electrode capillaries (*Ф* = 1.5 99 mm, wall thickness 0.178 mm, Hilgenberg) were filled with 3 M KCl, with tip openings of 0.4–1 MΩ. A USB-6221 DAQ device (National Instruments) and WinWCP (v5.5.3, Strathclyde University, United Kingdom) were used for data acquisition. Origin2020 Pro was used for data analysis. The laparotomy to obtain oocytes from *Xenopus laevis* was carried out in accordance with the principles of the Basel Declaration and recommendations of Landratsamt Wuerzburg, Veterinaeramt. The protocol under license #70/14 from Landratsamt Wuerzburg, Veterinaeramt, was approved by the responsible veterinarian.

### Patch-Clamp Experiments

Patch-clamp experiments were performed using a setup described in detail before (García-Martínez et al., 2015; Adam et al., 2018; Panzer et al., 2019). Measurements of the tandem proteins were performed with the lasers used previously (García-Martínez et al., 2015). For ApOps 2.0 constructs, the beam of a 532 nm DPSS laser (Changchun New Industries Optoelectronics) was coupled into a 400 µm light fiber that was coupled to a fiber-optic cannula (Thor labs, CFM14L10, *Ø*400 µm Core, 0.39 NA). Cells were illuminated directly in front of the fiber. For the measurement of dose dependency (0.02–84 mW mm^−2^), the laser intensity was set by means of neutral density (ND) filters. The composition of the standard patch-clamp solutions was described recently (Adam et al., 2018). For the identification of the transported ion species in the extracellular solution, NaCl was replaced with an equimolar concentration of sodium gluconate (absence of chloride). Similarly, in the pipette solution, NaCl was replaced with an equimolar concentration of CsCl. A voltage-step protocol was used for recording the *I*–*V* plots. The time between start of sweeps was set to 2 s (time without illumination: 1.9 s). Data were analyzed with ClampFit 10.7 software, Excel, and Origin Pro 2016 64Bit. The mean and standard deviation of the values obtained from different cells were calculated. Current values were normalized to the value obtained at a 0 mV clamp voltage in bath solution NaCl pH 7.4 and plotted against the applied membrane voltage. Action spectra were recorded and analyzed as described previously (García-Martínez et al., 2015).

## Results

### The Genome of *A. pullulans* Encodes for Three Rhodopsins

**FIGURE 1:**
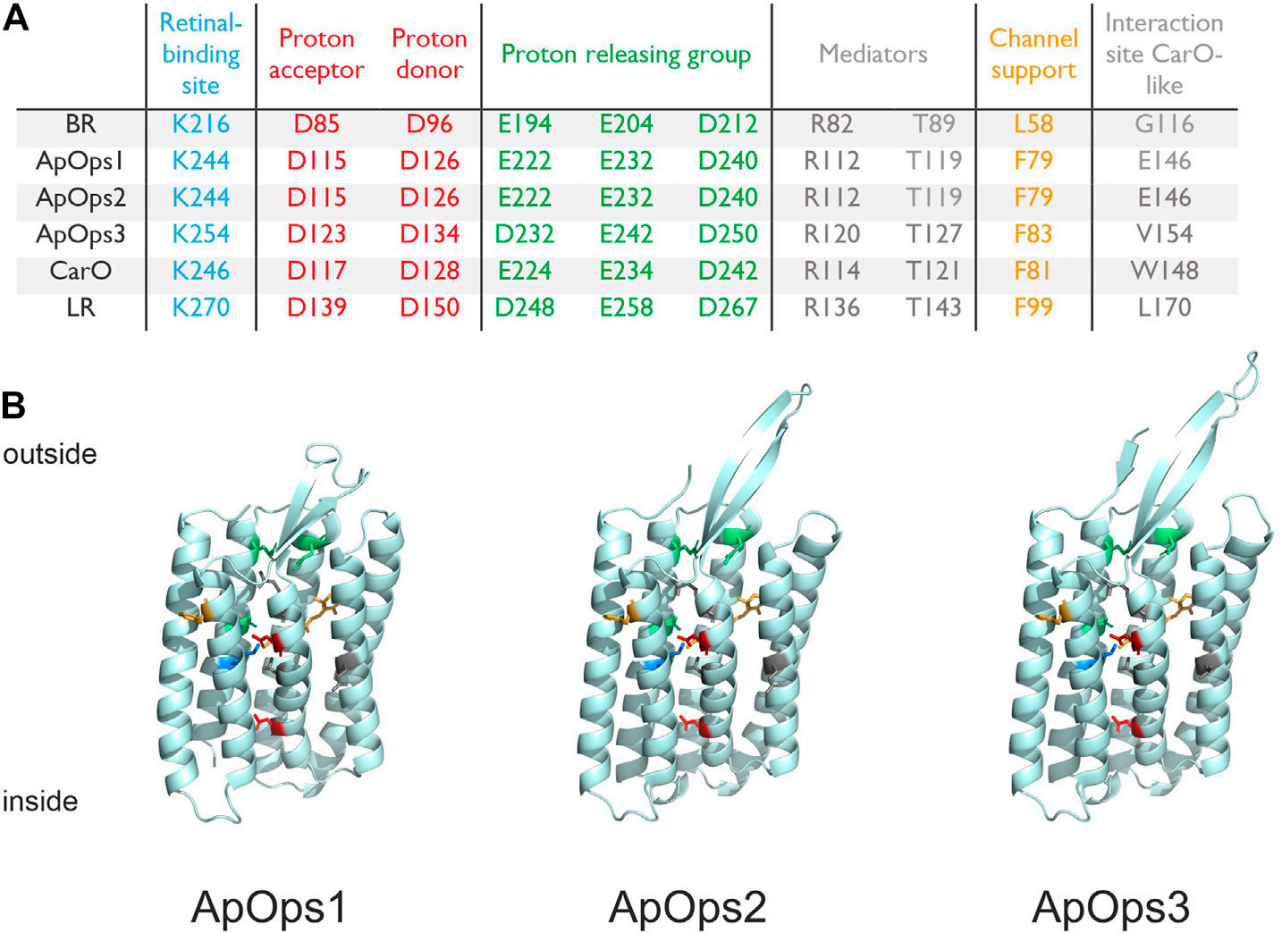
Conserved residues and homology models of *A. pullulans* rhodopsins. **(A)** Conserved residues of bacteriorhodopsin (BR), *A. pullulans* rhodopsin1 (ApOps1), ApOps2, ApOps3, *F. fujikuroi* CarO, and *L. maculans* rhodopsin (LR) are listed, showing the amino-acid sequence number as well as the respective residue in this position. **(B)** Model of the protein structures as indicated. Swissmodel was used to model the *A. pullulans* rhodopsins using the crystal structure of *Coccomyxa subellipsoidea* Rhodopsin CsR (6gyh.1.A) for ApOps1 and of LR (7bmh.1.A) for ApOps2 and ApOps3.

We BLAST-searched the genome of *Aureobasidium pullulans* (isolate EXF-150, CBS 100280, from the hypersaline waters of the Sečovlje solar saltern in Slovenia) (Gostinčar et al., 2014) and found three genes coding for putative opsins. The putative (rhod)opsins ApOps1 (gb|KEQ89910.1), ApOps2 (gb|KEQ89333.1), and ApOps3 (gb|KEQ87154.1) were identified by conserved residues that are typically present in microbial rhodopsins including the lysine in the seventh transmembrane domain providing the retinal binding site (Figure 1; complete sequence in Supplementary Figure S1). ApOps1 and ApOps2 share 59% sequence identities, and both are assigned to the clade of CarO-like rhodopsins, while ApOps3 is a representative of the LR-like rhodopsins (Adam et al., 2018). All three rhodopsins also show aspartate residues in the proton-acceptor position, homologous to BR(D85), as well as in the proton-donor position (a homologue of BR-D96). The proton-releasing group (BR: E194, E204, D212) consists of either two glutamate and one aspartate residues (ApOps1 and ApOps2) or one glutamate and two aspartate residues (ApOps3). While ApOps1 and ApOps2 show glutamate in the BR-G116 analogue position, which was considered as a hallmark of fungal rhodopsins (Fan et al., 2011), we find a valine in ApOps3 in this position. Overall, from the conserved residues, we would expect proton pump functions from all three *A. fumigatus* rhodopsins.

Using Swissmodel, we modeled the 3D structure of all three rhodopsins. All three rhodopsins consist of seven transmembrane domains. As observed in other fungal rhodopsins like CarO from *F. fujikuroi* or the rhodopsins from *U. maydis* also, all three ApOpsins show an extended extracellular loop between TM2 and TM3 which might play a role in the regulation of pump activity (Zabelskii et al., 2021). Modeling ApOps2 and ApOps3 to the crystal structure of LR (7bmh.1.A) revealed solid QMEANDisCo values of 0.71 ± 0.05 and 0.86 ± 0.05, respectively. Modeling of ApOps1 to LR (0.69 ± 0.05) revealed slightly smaller values than to *Coccomyxa subellipsoidea* Rhodopsin CsR (6gyh.1.A; 0.72 ± 0.06). The major difference observed in the two models of ApOps1 is the folding of the extracellular loop between TM2 and TM3, which was shown to interact with the N-terminus in LR (Zabelskii et al., 2021).

### Trafficking Signals Enhance Localization of *Aureobasidium* Rhodopsins in the Plasma Membrane

The expression of fungal rhodopsins in mammalian cells tends to be challenged by the fact that the trafficking is not optimally addressing the plasma membrane and some of the protein is sticking in the endoplasmic reticulum (Supplementary Figure S2).

**FIGURE 2:**
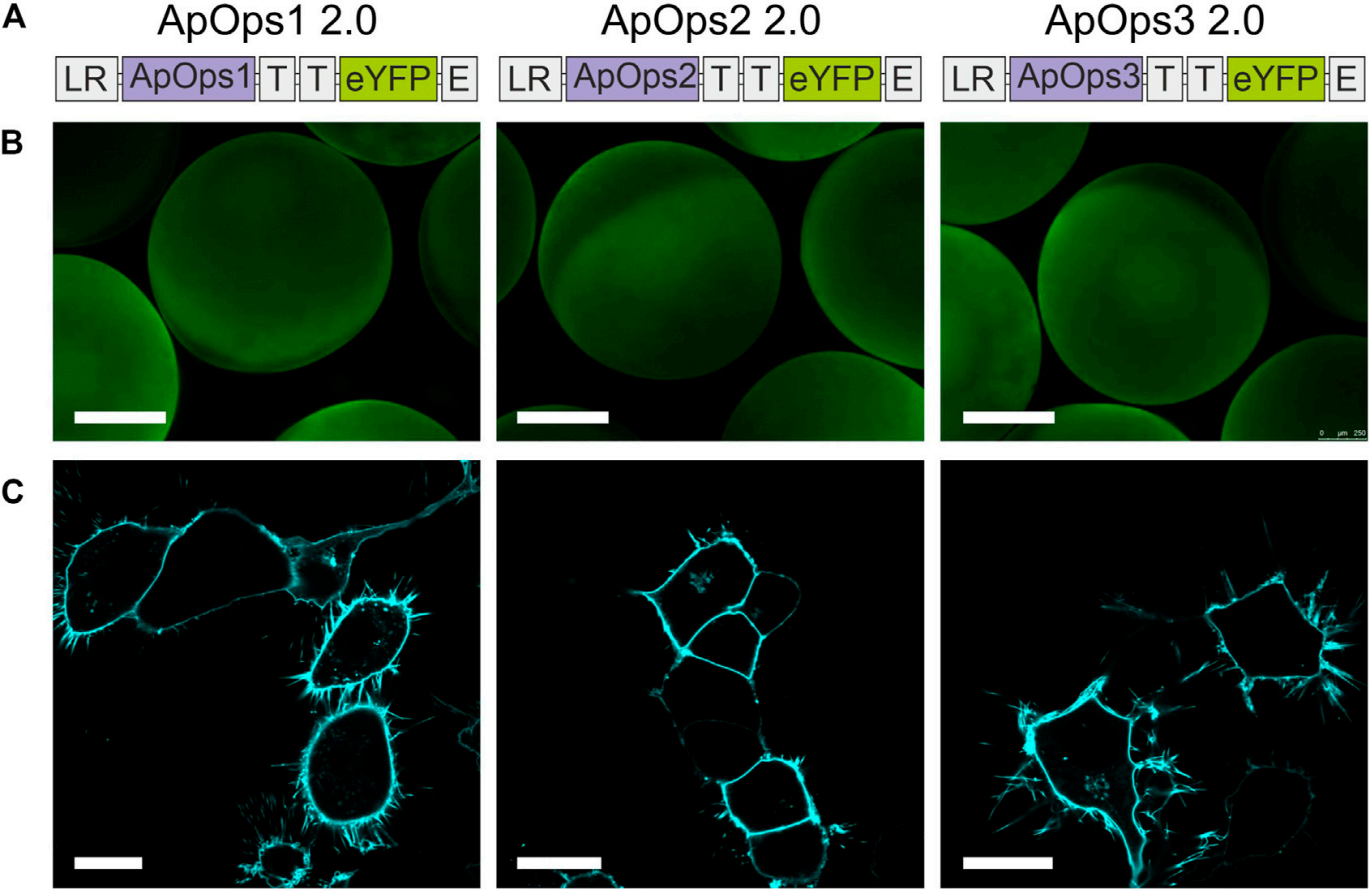
Heterologous expression of *Aureobasidium* rhodopsins in *Xenopus* oocytes and NG108-15 cells. **(A)** Rhodopsin expression and trafficking toward the plasma membrane were optimized by adding trafficking signals in the gene cassette as indicated. LR, the cleavable N-terminal signal peptide Lucy-Rho; T, the Golgi apparatus trafficking signal from Kir 2.1; and E, the endoplasmic reticulum export signal from Kir2.1. **(B)** Expressions of three optimized rhodopsins in Xenopus oocytes. Scale bar, 0.5 mm. **(C)** Confocal laser scanning images of NG108-15 cells expressing ApOps1-3 2.0. All three optimized rhodopsins were perfectly located in the plasma membrane and showed low/no expression in inner membranes. Scale bar, 20 µm.

We therefore added to our construct membrane trafficking factors from other membrane proteins, which were already successfully used in combination with other microbial rhodopsins for improved expression in plants (Zhou et al., 2021). The cleavable N-terminal signal peptide Lucy-Rho was linked to the N-terminus, the Golgi apparatus trafficking signal from Kir2.1 was used twice as a linker between the fungal rhodopsin and the eYFP tag, and the endoplasmic reticulum export signal from Kir2.1 was attached at the C-terminus (Figure 2A).

This modification of the membrane proteins led to improved expression and trafficking toward the plasma membrane (Figures 2B,C; Supplementary Figure S3A) and increased pump currents (Supplementary Figures S3B,C), whereas the physiological properties were unchanged (Supplementary Figure S3D). Also, the Lucy-Rho signal peptide improved the photocurrent of eArch3.0 [an improved version of Arch with maximal absorption around 530–550 nm (Mattis et al., 2011)] and *Coccomyxa subellipsoidea* rhodopsin (CsR), which was yet smaller than ApOps2 (Supplementary Figures S3B,C). Therefore, in the following, we present the electrophysiological data that were obtained with the expression-enhanced versions, called ApOps1 2.0, ApOps2 2.0, and ApOps3 2.0 hereafter.

### ApOps1, ApOps2, and ApOps3 Are Green-Light-Driven Ion Pumps

According to the structural models and the conserved residues, we expected all rhodopsins from *A. pullulans* to be proton pumps. To investigate this, we performed whole-cell patch-clamp experiments in the voltage-clamp mode at NG108-15 cells heterologously expressing the respective rhodopsin. All experiments were done at pH7.4 intra- and extracellular in the presence of sodium chloride and hence under conditions similar to those in previous investigations with other microbial rhodopsins (García-Martínez et al., 2015; Adam et al., 2018; Panzer et al., 2019).

**FIGURE 3:**
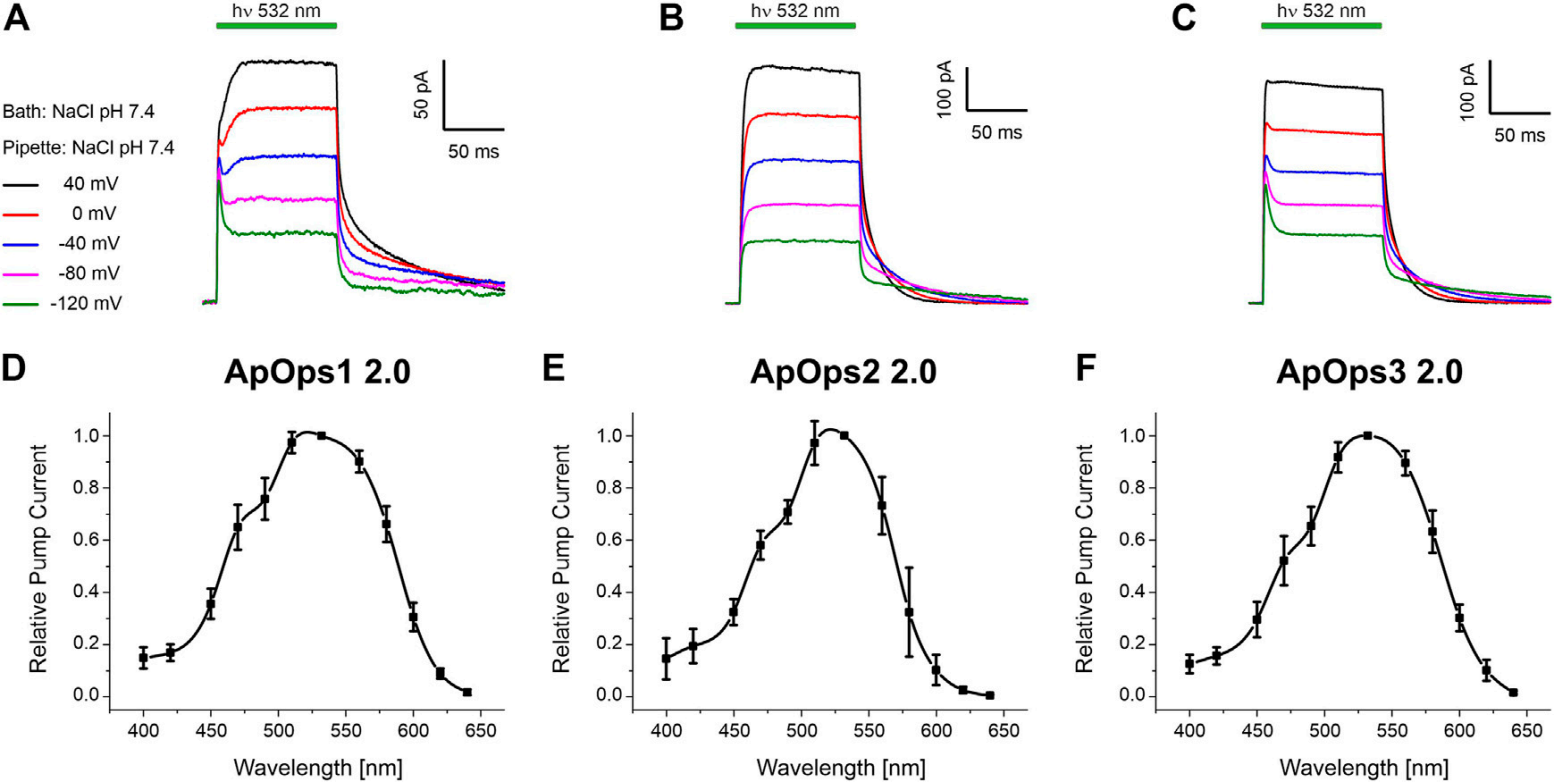
Whole-cell patch-clamp analysis of ApOps1 2.0 **(A,D)**, ApOps2 2.0 **(B,E)**, and ApOps3 2.0 **(C,F)** of *A. pullulans* in NG108-15 cells. **(A–C)** Voltage dependency of the green-light-induced pump signal. Note that the initial transient pump current is only pronounced in ApOps1 2.0 and ApOps3 2.0 at negative membrane potentials, while at depolarized membranes, the stationary current is dominating. In ApOps2 2.0, under these conditions, no initial transient peak is detectable. **(D–F)**. Action spectra of ApOps1 2.0 **(D)**, ApOps2 2.0 **(E)**, and ApOps3 2.0 **(F)**. The mean relative pump activity and standard deviation of *n* = 6 measurements as indicated are given in dependence of the wavelength used for excitation. All rhodopsins show the highest activation in the green spectral range.

Indeed, when illuminated with green light (DPSS-laser, 532 nm), all rhodopsins responded with a typical pump signal, similar to those observed with other fungal rhodopsins like CarO (García-Martínez et al., 2015; Adam et al., 2018) or UmOps1 and UmOps2 (Panzer et al., 2019) (Figures 3A–C). Hence, ApOps1, ApOps2, and ApOps3 are light-gated ion pumps. We also analyzed the voltage dependency of the rhodopsins, revealing that all rhodopsins are still active at negative membrane potentials. Nevertheless, as expected from the increasing gradient, the pump activity decreased with more negative membrane potentials. Notably, the characteristic initial peak that is well pronounced in many microbial proton pumps like BR and CarO was only present in ApOps1 and ApOps3, especially at more negative membrane potentials, whereas at more positive voltages, the stationary currents were dominating. Interestingly, at depolarized membranes, ApOps1 reached its maximal activity late, after about 20–30 ms (Figure 3A).

All three rhodopsins show a biexponential current decay upon light-off, exhibiting a fast and a slow time constant. The fast time constants fall into the lower single-digit range, and the slow ones fall in the double and lower three-digit range. The slow time constants show a clear voltage dependency. Concerning the mean slow time constant, ApOps1 exhibited much higher values than ApOps2 and ApOps3, suggesting a slow photocycle [at 0 mV 72 ± 16 ms (ApOps1) vs 19 ± 2.9 ms (ApOps2) and 20.2 ± 2.6 ms (ApOps3); Supplementary Figure S4].

Fungal rhodopsins typically are supposed to perceive green light. To investigate if this is also the case for the *Aureobasidium* rhodopsins, we analyzed the spectral range promoting maximal pump activity by recording the action spectrum. All rhodopsins yielded bell-shaped action spectra ranging from 400 to 640 nm (Figures 3D–F) with the maximal pump activity in the green spectral range (excitation with 532 nm).

Of note, the pump activity of these rhodopsins, especially of ApOps2 and ApOps3, was relatively high in comparison to other fungal rhodopsins, reaching mean current densities of 4.5 ± 1.2 pA/pF (*n* = 16, ApOps1 2.0), 5.4 ± 4.8 pA/pF (*n* = 13, ApOps2 2.0), and 7.3 ± 3.2 pA/pF (*n* = 14, ApOps3 2.0) at 15 mW mm^−2^. However, the highest current densities were observed with ApOps2 2.0, sometimes reaching values above 15 pA/pF.

The number of activated pumps can be calculated from the amplitude of the light-induced pump-current I_stat_ and the slow time constant of current decay τ_slow_ under saturating conditions and assuming that one charge is transported per photon. The products of I_stat_ and τ_slow_ divided by the elementary charge (*e* = 1.6 * 10–^19^ As) and the cell surface area [obtained from the membrane capacitance divided by the NG108-15-specific membrane capacitance of 1.72 ± 0.14 μF/cm^2^ (Feldbauer et al., 2016)] yield 10.8 ± 7.2 * 10^3^ pumps/µm^2^ (ApOps1 2.0, *n* = 5), 7.4 ± 5.7 * 10^3^ pumps/µm^2^ (*n* = 5, ApOps2 2.0), and 4.9 ± 2.9 * 10^3^ pumps/µm^2^ (*n* = 8, ApOps3 3.0), suggesting that ApOps2 and ApOps3 are more potent pumps reaching higher pump activities with a lower expression rate.

For the comparison of protein functions independent of the expression level, we used a genetic tandem cassette combining two rhodopsins in one functional protein (Kleinlogel et al., 2011; Feldbauer et al., 2016; Vierock et al., 2020). This protein combines an N-terminal rhodopsin (here, ChR2) *via* a fluorescent protein and the beta-subunit of the H^+^/K^+^-ATPase with the second rhodopsin (the respective fungal rhodopsin) in the C-terminal position (Supplementary Figure S5A). We measured the fungal rhodopsin currents (561 nm = low activation of ChR2) at 0 mV and normalized it to the ChR2-currents (473 nm) at –120 mV (Supplementary Figures S5B,C). With this stoichiometric comparison in the tandem protein, the pump activity of ApOps2 was about 4 times higher than that of ApOps1 (Supplementary Figure S5D). This is in accordance with the faster time constants observed in ApOps2 (Supplementary Figure S4) and shows once more that this relatively high pump activity was not (only) due to an expression effect but an intrinsic feature of this fungal rhodopsin. Indeed, in this comparison, MAC, the fungal rhodopsin currently used most in optogenetic applications, showed 35% less activity than ApOps2 under the same conditions (Supplementary Figure S5D). High pump activity is directly coupled to high energy consumption. In accordance, we found that ApOpsins are saturating at very high light intensities higher than the maximal solar radiation (Supplementary Figure S6).

### All Three *Aureobasidium* Rhodopsins Transport Solely Protons

**FIGURE 4:**
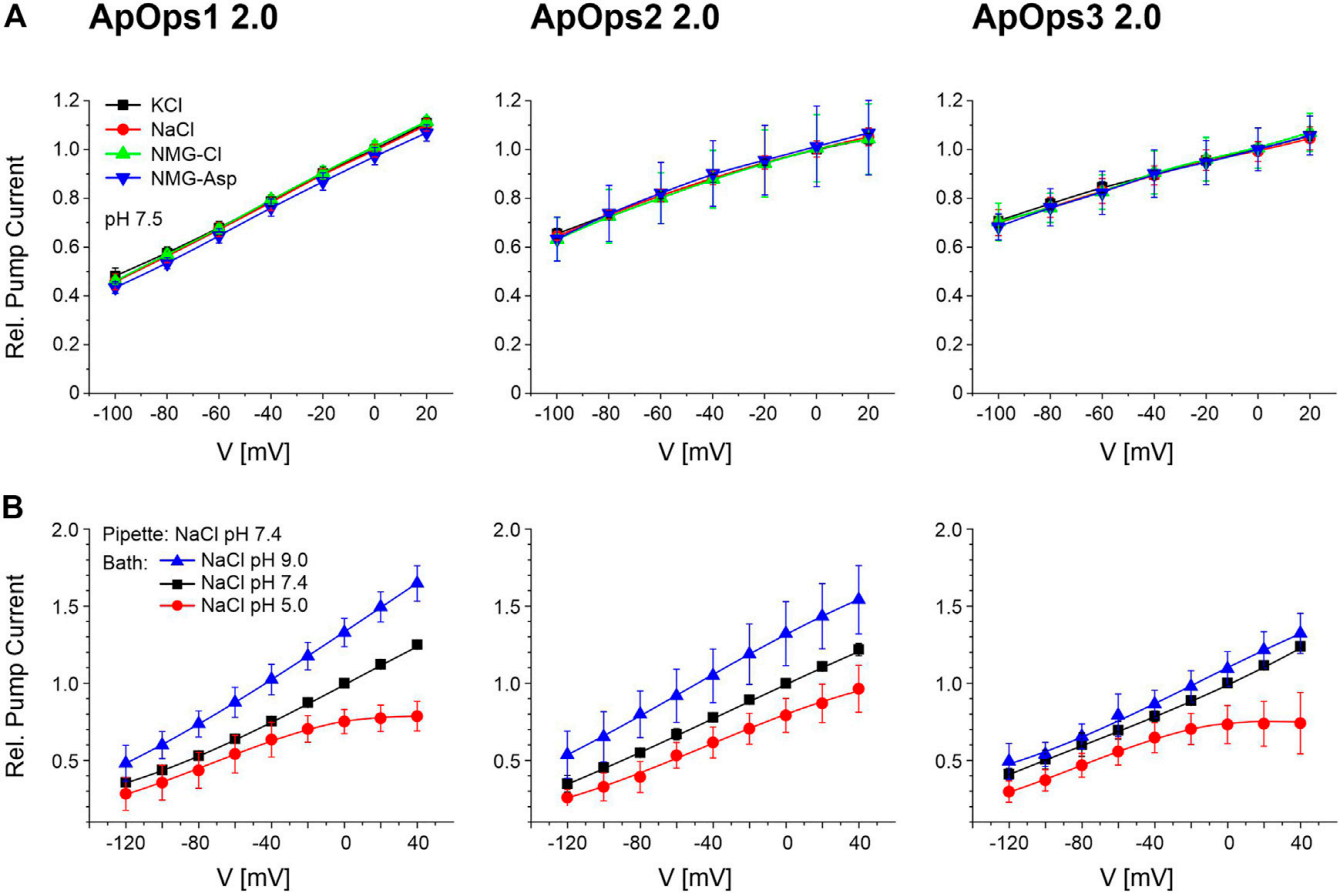
Current–voltage relation of ApOps1 2.0, ApOps2 2.0, and ApOps3 2.0 in different extracellular solutions as indicated. **(A)** Relative photocurrents of ApOps1-3 2.0 in different pH 7.5 buffers comprising 115 mM of the respective salt as indicated measured by the TEVC method in the *Xenopus* oocyte normalized to the value obtained in KCl at the holding potential of 0 mV (mean ± SD, *n* ≥ 6). **(B)** pH dependency of the *A. pullulans* rhodopsin green-light-induced pump activity in NG108-15 cells as indicated. Relative pump activity of ApOps1 2.0, ApOps2 2.0, and ApOps3 2.0 in the range of +40 to –120 mV after excitation with a 532 nm laser normalized to the value obtained in bath solution NaCl pH 7.4 at a 0 mV clamp voltage [mean +SD; *n* = 5 (ApOps1 2.0), *n* = 7 (ApOps2 2.0), and *n* = 8 (ApOps3 2.0)]. Note that pump activity is not further increasing at positive voltages in an acid environment (pH 5) for ApOps1 2.0 and ApOps3 2.0.

To investigate if ApOps1-3 are really proton pumps, we first analyzed the pump currents in different buffers of the same pH by TEVC with *Xenopus* oocytes. The photocurrents of ApOps1-3 were similar in different buffers of the same pH 7.5 (Figure 4A). We then further tested the pH dependency of the pump activity in NG108-15 cells, where the intracellular pH can be controlled. An outward-directed proton pump should be challenged by an acid extracellular environment and negative membrane potentials (Figure 4B). Indeed, all three rhodopsins showed this expected behavior, with the lowest pump activity at negative voltages in pH 5. In contrast, the highest pump activity was observed at pH 9 and positive voltages. This is in accordance with the hypothesis that these proteins act as proton pumps. Interestingly, for ApOps1 and ApOps3, we observe an I-V curve non-linear at positive membrane potentials (Figure 4B).

To further confirm the proton pump activity in NG108-15 cells, we replaced either extracellular chloride with gluconate or intracellular sodium with cesium in the whole-cell patch-clamp measurements as besides protons, potentially, chloride or sodium is a potential target for the charge transfer by the ApOps proteins as well. The resulting I-V relationship was similar to the I-V curve observed with NaCl (Supplementary Figure S7; Figure 4B), with the only exception that the saturation effect observed from ApOps1 and ApOps3 in sodium chloride was absent under these conditions, suggesting a modulating role of sodium under acidic conditions. Furthermore, when using gluconate, a slightly enhanced pump current could be measured. This issue derives from the “weak organic acid (WOA) effect,” which could be shown previously for other fungal rhodopsins (Garcìa-Martìnez et al., 2015; Adam et al., 2018; Panzer et al., 2019) dealing with the fact that rhodopsins show increased pump activity in the presence of extracellular weak organic acids.

While for ApOps1 2.0 and ApOps2 2.0 the current densities were similar, in contrast, for ApOps3 2.0, we observed a significant decrease in pump activity in the presence of cesium/absence of sodium (Supplementary Figure S7).

### Site-Directed Mutagenesis Change Pumping Behavior Toward Channels

**FIGURE 5:**
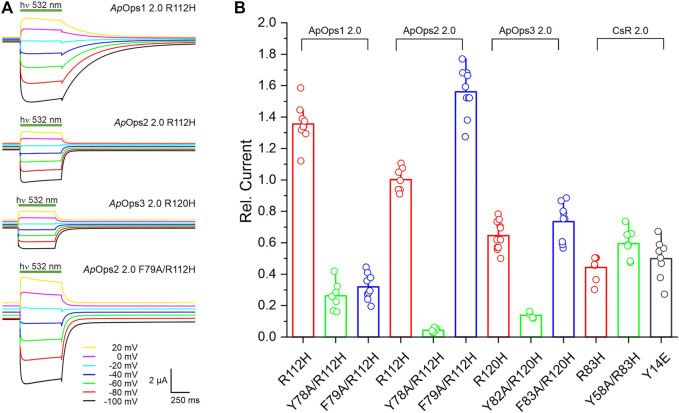
Analysis of ApOps1-3 2.0 proton channel mutants. Introduction of two point mutations by site-directed mutagenesis leads to altered behavior of the protein, changing from the proton pump to proton channel. **(A)** Typical proton channel current traces upon illumination with green light (532 nm DPSS laser, 7 mW mm^−2^) in the Ori standard buffer (with 2 mM Ca^2+^, pH = 7.6) of different ApOps1-3 2.0 mutants as indicated. **(B)** Comparison of the relative proton channel current obtained from different proton pump rhodopsin mutants in the Ori standard buffer (with 2 mM Ca^2+^, pH = 7.6) at the holding potential of –100 mV. Current was normalized to the value of ApOps2 2.0 (R112H).

ApOps2 and ApOps3 showed very intense pump activity, making them potentially superior optogenetic tools for membrane voltage and pH regulation. Besides proton pumps, proton-specific channels are of interest for the regulation of pH by light as well. We therefore wanted to test if the *Aureobasidium* proton pumps could be altered by mutagenesis to proton channels. In our previous studies with a *Fragilariopsis* proton pump rhodopsin, a random mutation of arginine (R) to histidine (H) happened during the cloning process. Since the R position is mostly conserved in the transmembrane helix 3 of different rhodopsins and it is known that it is of importance for the proton transfer, we decided to keep the wrong clone/mutant for a measurement. We found it to be a light-gated proton channel (unpublished, PhD thesis of S. Gao). We used site-directed mutagenesis to introduce the similar mutation R112H to ApOps2 and also the corresponding mutations in ApOps1 and ApOps3 (Figure 5A). We found that this single mutation turns ApOps1-3 into light-gated proton channels (Figure 5A). Interestingly, ApOps1 with the least proton pump current showed the largest channel current after mutation (Figure 5B). From the modeled structure, we thought that Y78 or F79, corresponding to Y57 in CsR, might participate in conformational modification of the proton-transport pathway. Introducing an additional mutation F79A indeed further increased the proton channel current of ApOps2 R112H (Figure 5A). A single F79A mutation will not change the proton pump character but only influence the photocurrent slightly (Supplementary Figure S8). ApOps2 2.0 F79A/R112H yielded the largest proton channel current among the tested ApOps1-3 mutants (Figure 5B). The ApOps2 2.0 F79A/R112H mutant showed about a 3-fold higher proton channel current than the corresponding CsR mutants and the previously published CsR Y14E mutant, whose reversal potential did not meet requirements for a pure proton channel (Fudim et al., 2019). In general, protein expression and membrane insertion in the mutants were similar to the wild type (Supplementary Figure S9A).

**FIGURE 6:**
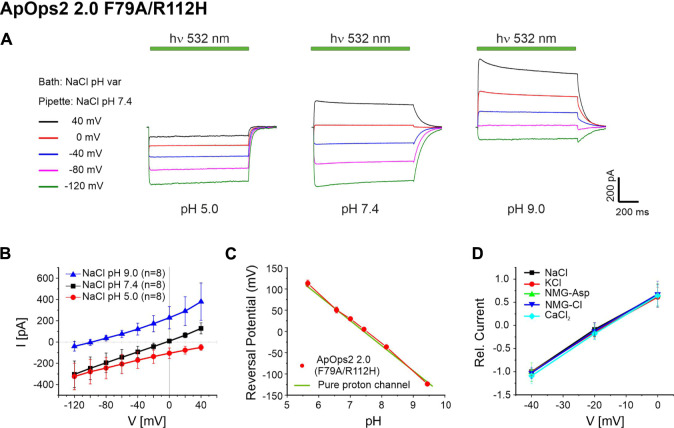
Electrophysiological analysis of ApOps2 2.0 F79A/R112H. **(A)** Typical current trace of ApOps2 2.0 F79A/R112H in NG108-15 cells upon illumination with green light (532 nm DPSS laser, 15 mW mm^−2^) at different pH values as indicated in whole-cell patch-clamp experiments. Intracellular solution: NaCl pH 7.4, extracellular solution: NaCl, different pH. **(B)** I-V plot of the absolute light-induced current (mean + SD of *n* = 8 NG108-15 cells). **(C)** Comparison of the calculated reversal potential of a pure proton channel at different pH with the reversal potential measured in *Xenopus* oocytes (mean + SD, *n* ≥ 3). **(D)** Ion selectivity analysis of ApOps2 2.0 F79A/R112H in *Xenopus* oocytes in different buffers as indicated (mean + SD, *n* ≥ 11). Current was normalized to the absolute value obtained in 115 mM NaCl buffer (with 2 mM Ca^2+^, pH = 7.6) at the holding potential of −40 mV. All buffers contained 115 mM of the respective salt (except 20 mM CaCl_2_).

Due to the promising intense pump activity, we further investigated the ApOps2 2.0 F79A/R112H mutant (Figure 6). In NG108-15 cells, this mutant showed good expression (Supplementary Figure S9B) and light-gated proton channel activity (Figure 6A) that was strongly dependent on the extracellular pH (Figures 6A,B). In *Xenopus* oocytes, we showed that the reverse potentials measured for ApOps2 2.0 F79A/R112H are similar to the reversal potentials calculated for protons using the Nernst equation with E_s_ = 58.17*(pHi-pHe), and pHi and pHe represented intracellular and extracellular pH, respectively (Figure 6C). pHi was regarded as 7.43 (Cicirelli et al., 1983). In accordance, testing the ApOps2 2.0 F79A/R112H double mutant in different extracellular buffers confirmed the current specificity for protons (Figure 6D).

## Discussion

Optogenetic applications benefit from a broad spectrum of photosensitive proteins with different characteristics that can be chosen in dependence of the respective scientific aim (Adamantidis et al., 2015). Microbial rhodopsins are used frequently in pathophysiological investigations since the very beginning (Bamann et al., 2010). Here, we analyzed three fungal rhodopsins from *A. pullulans*, ApOps1, ApOps2, and ApOps3, that all represent green-light-driven proton pumps (Figure 3). Specificity of proton transport was deduced from the conserved residues and structural similarity with LR (Zabelskii et al., 2021) (Figure 2) and proven by pH dependency (Figure 4) as well as unchanged pump activity (Figure 4) in the absence of extracellular chloride (replacement with glutamate/aspartate), sodium (replacement with potassium/NMG), or intracellular sodium (replacement with cesium; Supplementary Figure S7), with exception of ApOps3. For the latter, the pump activity decreased in the absence of sodium. The high similarity with the proton pump LR and the absence of residues required for sodium transport in other microbial rhodopsins indicate ApOps3 to be a proton pump. One may thus conclude that sodium is boosting up and/or cesium is reducing the pump activity.

As ApOps2 and ApOps3 are very potent pumps reaching unusually high pump activities with values above 5 pA/pF, we expect them to be promising candidates for future optogenetic applications. For comparison, the current intensity of CarO from *F. fujikuroi* was 2.2 ± 0.6 pA/pF, and in UmOps1 and UmOps2 of *U. maydis*, even current densities below 0.1 pA/pF were reached (Panzer et al., 2019). However, when comparing the data, one should take into account that using the 2.0 cassette strongly improved the trafficking toward the plasma membrane for ApOps1 and ApOps2 (Figure 1, Supplementary Figure S2) and thus likely will have increased the amount of rhodopsins accessible during electrophysiological measurements.

In optogenetic applications, high turnover rates of microbial rhodopsins are sought after (McIsaac et al., 2015). The slow time constants of ApOps2 and ApOps3 are with ∼20 ms in a similar range to other proton pumps. Despite the relatively slow time constants observed in ApOps1 (ca. 72 ± 16 ms at 0 mV), the mean current densities obtained with this rhodopsin were still relatively high and similar to the mean current densities of ApOps2 (Supplementary Figure S7).

A slow-cycling pump is expected to reveal less pump activity than a fast-cycling one. Thus, the high current densities obtained with ApOps1 are expected to be at least partly due to the expression level. Indeed, when comparing the number of active rhodopsins, we found the highest amount of proteins for ApOps1 2.0 (ca. 11,000 rhodopsins per µm^2^) in comparison to ApOps2 2.0 (7,400) and ApOps3 2.0 (4,900). The rhodopsin density was in a similar range as previously described for HEK293 cells expressing ChR2-eYFP (Zimmermann et al., 2008). Another support for ApOps2 being a more potent proton pump than ApOps1 was obtained from the comparison of the photocurrent amplitudes of the fungal rhodopsins independent of their expression level (ChR2-eYFP-βH-[fungal-rhodopsin]-tandems; Supplementary Figure S5). Accordingly, in single experiments, ApOps2 2.0 showed the highest current densities of all fungal rhodopsins, reaching values of above 15 pA/pF, while some cells exhibited very low expression levels. Therefore, the mean current density of ApOps2 2.0 is lower than that of ApOps3 2.0.

Interestingly, at high light intensities under acidic conditions, the release of protons seems to be saturated in ApOps1 2.0 and ApOps3 2.0 as no further increase of the relative pump activity can be observed at positive membrane potentials (Figure 4B). In the absence of intracellular sodium/presence of cesium, the pump activity of ApOps3 2.0 is decreased and in accordance with the before-mentioned saturation effect cannot be observed under these conditions any more (Supplementary Figure S7).

The 2.0 expression cassette (Zhou et al., 2021) improves the membrane insertion by far without detectable effects on the physiological characteristics of the respective rhodopsin while increasing the current densities due to higher numbers of proteins in the plasma membrane (Figure 1; Supplementary Figure S2 and Supplementary Figure S3). Thus, using this 2.0 cassette could be of general interest for optogenetic applications but also for the investigation of fungal rhodopsins like UmOps2 that natively are trafficked to the vacuole (Panzer et al., 2019). However, manipulation of the protein sequence should be done carefully as modification of the N-terminus in fungal rhodopsins might alter the protein function as this domain may be involved in regulation of protein functions (Zabelskii et al., 2021).

Furthermore, a potent proton pump is expected to provide an optimized water network that may be diverted by mutagenesis to provide a highly permeable proton-selective channel. The position BR-R82 has been shown to play an important role in the proton release to the extracellular bulk solvent (Ernst et al., 2014), and replacement of Arg with Gln in CsR R83 led to a channel-like behavior (Vogt et al., 2015). Indeed, we succeeded in altering the protein function of ApOps2 2.0 by introducing the point mutations F79A and R112H. In contrast to native channelrhodopsins, these artificial leaky proton pumps are highly selective for protons but are not transporting other cations, which might be of interest for several approaches. This mutation was functional not only in *Xenopus* oocytes but also in NG108-15 cells (Figures 5, 6).

Thus, *Aureobasidium* benefits us with potent green-light-gated pumps, partly providing higher activity than LR that was used in several optogenetic applications (Chow et al., 2010). Such tools may be of importance for the modulation of pH in the analysis of pH regulation in cells (Rost et al., 2015). In addition, fungal rhodopsins providing pump and channel characteristics might be of interest for future optogenetic applications of fungi, a so far unexplored scientific field.

Besides optogenetics, we are interested in the biological function of fungal rhodopsins and the potential role of these rhodopsins in *Aureobasidium*. Interestingly, *Aureobasidium* provides three pumping rhodopsins of the CarO and LR type but not the slow-cycling Nop1 type (Adam et al., 2018; Wang et al., 2018). Why the fungus needs these light-driven pumps is not clear; however, it is likely that these rhodopsins contribute to maintenance of the proton-motive force that normally would require consumption of (more) ATP. Thus, the fungus would use light to spare ATP for other approaches. It should be taken into account that *Aureobasidium* is an extremophilic fungus that is exposed to very high salt concentrations. In order to survive these conditions, entering sodium has to be removed from the cytosol. Therefore, the decrease in absolute pump currents we observe in ApOps3 in the absence of sodium with intracellular CsCl might be of biological importance. We may interpret this observation as a requirement of the presence of sodium for higher pump activity of ApOps3. To maintain the cytosolic sodium level relatively low, the fungus will use sodium-proton exchangers, antiporters, that are driven by protons. Thus, increasing the driving force for sodium extrusion by light might support the fungus in persisting high salt concentrations without spending much energy in building up the proton motive force, which is normally maintained under consumption of ATP. From the biological point of view, ApOps3 may be more active, when the fungus is exposed to high extracellular sodium concentrations. This context might be investigated in the future.

The absence of saturation even at very high light intensities that are more than 10-fold above typical sun radiation (∼1 mW mm^−2^) is striking, even for extremophils that can be exposed to very strong radiation. Nevertheless, the absence of saturation at a value of 20 mW mm^−2^ was previously observed in eArch3.0, a proton pump from the halophilic archaeon *Halorubrum sodomense* (Mattis et al., 2011). It is questionable if such high light concentrations are ever obtained in the rhodopsin environment under natural conditions. In this context, one should investigate in the future if the fungus maintains optical systems like in *Chlamydomonas rheinhardtii* that concentrate the light and focus it onto the receptors, which would lead to much higher light intensities (Boyd et al., 2011). On the other hand, fungi are full of carotenoids, and it cannot be excluded that under natural conditions beside retinal, other chromophores harvest additional photons as in Xanthorhodopsins (Balashov and Lanyi, 2007). When interpreting the protein function, one should also consider that the membrane environment and membrane tension are quite different in mammalian cells compared to those in the black yeast when exposed to very high salt concentrations.

## Data Availability

The raw data supporting the conclusion of this article will be made available by the authors, without undue reservation.
