# Supplementary material for: Modified Rhodopsins From Aureobasidium pullulans Excel With Very High Proton-Transport Rates
Source: Front Mol Biosci. 2021 Nov 1;8:750528. doi: 10.3389/fmolb.2021.750528 (PMC8591190; doi:10.3389/fmolb.2021.750528)
Supplement: Supplementary file 1 [file DataSheet1.pdf]

Supplementary Information

Modified rhodopsins of *Aureobasidium pullulans* excel with very high proton-transport rates

Sabine Panzer[1, †], Chong Zhang[2 †], Tilen Konte[3], Celine Bräuer[1], Anne Diemar[1], Parathy Yogendran[1], Jing Yu-Strzelczyk[2], Georg Nagel[2], Shiqiang Gao[2], Ulrich Terpitz[1,*]

[1] Department of Biotechnology and Biophysics, Theodor-Boveri-Institute, Julius Maximilian University of Wuerzburg, Biocenter, Wuerzburg, Germany

[2]Physiological Institute, Department of Neurophysiology, Julius Maximilian University of Wuerzburg, Wuerzburg, Germany.

[3] Institute of Biochemistry, Faculty of Medicine, University of Ljubljana, Ljubljana, Slovenia

[†]These authors have contributed equally to this work and share first authorship

```
                             ....|....| ....|....| ....|....| ....|....| ....|....|
                                     10         20         30         40         50
Fus.fuj.CarO|emb|CAD97459    ---------- --------MA DHLYARKNDA LNVNPDIVNG QRSDINITVR
Aur.pull.ops1|gb|KEQ89910    ---------- ---------- MSWLEKRNDA IQVNPNTQNN KHVDIAITVR
Aur.pull.ops2|gb|KEQ89331    ---------- ---------- MDFLQKRNDA LNVNPNMVNG KSSDIAITVR
Aur.pull.ops3|gb|KEQ87154    ---------- ------MIVD PVEAFKATSS VAPIPTVVPS LPEYETVTET
Lep.mac.ops1 |gb|AAG01180    MIVDQFEEVL MKTSQLFPLP TATQSAQPTH VAPVPTVLPD TPIYETVGDS
Clustal Consensus                                             :   *    .       :

                             ....|....| ....|....| ....|....| ....|....| ....|....|
                                     60         70         80         90        100
Fus.fuj.CarO|emb|CAD97459    GSDWYWAVCA VMTVSTFAFL GLGMRKPRTD RIFHYITAGI TMIASIAYFT
Aur.pull.ops1|gb|KEQ89910    GSDFYFAICA VMGFVALGVM AASAMKPRTD RIFFYITAAI NTTACIAYFA
Aur.pull.ops2|gb|KEQ89331    GSDWYWAVCA VMTMATFVFL GLGVTKPRQH RVFHYITAAI TMVAAIAYFS
Aur.pull.ops3|gb|KEQ87154    GTRALWAVFV LMLLSMIVFV GLSWTVPISK RLYHVVTTLI VTFASLSYFA
Lep.mac.ops1 |gb|AAG01180    GSKTLWVVFV LMLIASAAFT ALSWKIPVNR RLYHVITTII TLTAALSYFA
Clustal Consensus            *:   :.: . :* .    .  . .   *    *::. :*: *    *.::**:

                             ....|....| ....|....| ....|....| ....|....| ....|....|
                                    110        120        130        140        150
Fus.fuj.CarO|emb|CAD97459    MASNLGWTPI AVEFQRSNHR VAG----IYR EIFYARYIDW FLTTPLLLTD
Aur.pull.ops1|gb|KEQ89910    MGSNLGWTPI DVEWQRTWSQ VAG----VNR EVFYVRYIDW FVTTPLLLMD
Aur.pull.ops2|gb|KEQ89331    MASNLGWTPI DVEFQRNDPE VRG----INR EIFYVRYIDW FITTPLLLLD
Aur.pull.ops3|gb|KEQ87154    MATGHGISYH RTTVTDSHRH VPDTTHDVYR QVYWARYVDW SLTTPLLLLD
Lep.mac.ops1 |gb|AAG01180    MATGHGVALN KIVIRTQHDH VPDTYETVYR QVYYARYIDW AITTPLLLLD
Clustal Consensus            *.:. * :            . * .    : * ::::.**:**  :****** *

                             ....|....| ....|....| ....|....| ....|....| ....|....|
                                    160        170        180        190        200
Fus.fuj.CarO|emb|CAD97459    LLLTAGMPWP TVLWVILVDW VMIVTGLVGA LVKS--SYKW GYFAFGCAAL
Aur.pull.ops1|gb|KEQ89910    LLLTAGLPWP TILWTIFLDE VMIVTGLVGA LVKS--RYKW GFWTFGTVAM
Aur.pull.ops2|gb|KEQ89331    LMLTAAMPWP TILFIILVDE VMIVTGLVGA LVRS--SYKW GYFVFGCAAL
Aur.pull.ops3|gb|KEQ87154    LALLAGLSGG HILLAIVADV IMVLTGLFAA YGTEGTPQKW GWYAIACIAY
Lep.mac.ops1 |gb|AAG01180    LGLLAGMSGA HIFMAIVADL IMVLTGLFAA FGSEGTPQKW GWYTIACIAY
Clustal Consensus            * * *.:.    ::  *. *  :*::***..*    .    ** *::.:.  *

                             ....|....| ....|....| ....|....| ....|....| ....|....|
                                    210        220        230        240        250
Fus.fuj.CarO|emb|CAD97459    AYIVYVLAWE ARLHAKHVGP DVGRTFVMCG SLTAVVWILY PIAWGVCEGG
Aur.pull.ops1|gb|KEQ89910    FAIFWNLAVE GRKHAKHLGS DIARTYTICG CLTLFIWLCY PICWGVSEGA
Aur.pull.ops2|gb|KEQ89331    AYVVWVLVWE GRRHANVLGR DVGKAFTLCG SLTTFLWILY PVAWGICEGG
Aur.pull.ops3|gb|KEQ87154    LVVIWMLAVH GRANAMAKGG KVGKFFASIA GFTLVIWTIY PIVWGVADGS
Lep.mac.ops1 |gb|AAG01180    IFVVWHLVLN GGANARVKGE KLRSFFVAIG AYTLILWTAY PIVWGLADGA
Clustal Consensus              :.: *. . .  :*   *  .:   :.   .   * .:*  * *: **:.:*.

                             ....|....| ....|....| ....|....| ....|....| ....|....|
                                    260        270        280        290        300
Fus.fuj.CarO|emb|CAD97459    NLIAPDSEAV FYGILDLIAK PVFGALLLWG HRNIDPARLG LRIRDIDERI
Aur.pull.ops1|gb|KEQ89910    NVIPPDSEAV FYGVLDFLAK PVFSIALIIG HWNINPGRMG LKLRDYDEDP
Aur.pull.ops2|gb|KEQ89331    NIISPDSEAV FYGILDLLAK PVFGALLIWG HRGIDPARLG LYIHDYDEKD
Aur.pull.ops3|gb|KEQ87154    RKMSVDQEII AYAVLDLLAK PVFGAWLLFT HQSMPETQVE LGGFWTHGVS
Lep.mac.ops1 |gb|AAG01180    RKIGVDGEII AYAVLDVLAK GVFGAWLLVT HANLRESDVE LNGFWANGLN
Clustal Consensus            . :  * * :  *.:**.:**  **.  *:   * .:    :  *     .

                             ....|....| ....|....| ....|....| ..
                                    310        320        330
Fus.fuj.CarO|emb|CAD97459    FPDGPNNKVA SGHGARNDTA TASGSNVNPN A-
Aur.pull.ops1|gb|KEQ89910    DYFGPKNGAE AAKERSNGSS SGVDGGA--- --
Aur.pull.ops2|gb|KEQ89331    PAVKDKVGAP GPNVHPNSNN GVATNGQTAE TV
Aur.pull.ops3|gb|KEQ87154    SEGAIRVGED DEGA------ ---------- --
Lep.mac.ops1 |gb|AAG01180    REGAIRIGED DGA------- ---------- --
```

Supplementary Figure 1. Amino acid alignment of the three *Aureobasidium* rhodopsins in comparison with *Fusarium fujikuroi* CarO and *Leptosphaeria maculans* LR (Mac) using ClustalW analysis. Some important conserved residues are highlighted in colours and bold letters.

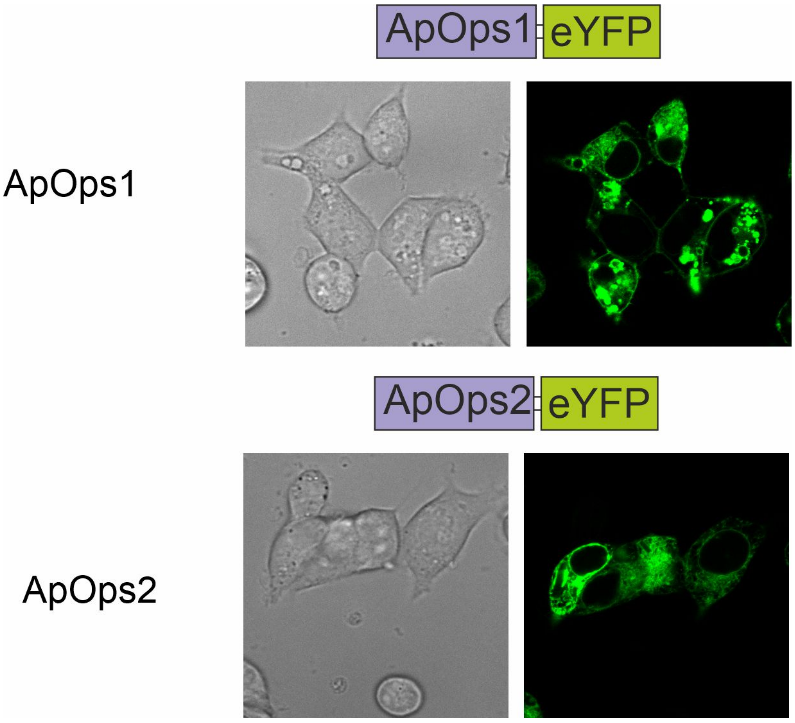

Supplementary Figure 2. Expression of ApOps1 and ApOps2 in NG108-15 cells without additional trafficking signals (2.0 –cassette). Note that a prominent percentage of the fluorescence is recorded from intracellular membrane systems that are not accessible to the patch-clamp technique in whole-cell configuration.

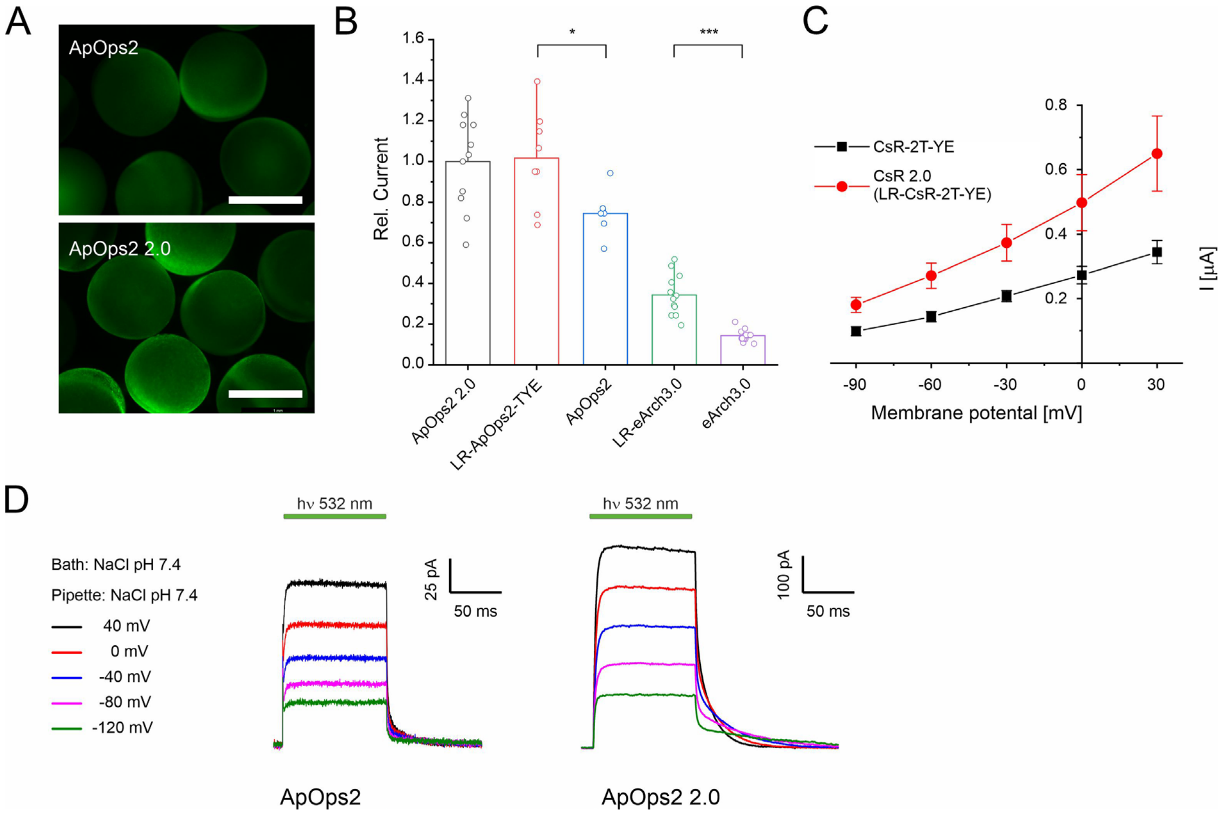

Supplementary Figure 3. Influence of membrane trafficking signals on the electrophysiological behaviour of microbial rhodopsin. A. Expression of ApOps2 in *Xenopus oocytes* with (lower) or without (upper) additional trafficking-improvement motifs. Scale bar , 1 mm. B. Comparing different modifications of ApOps2 and a widely used proton pump eArch3.0 after expression in *Xenopus* oocytes. TEVC experiment was performed in Ori standard buffer (2mM Ca^{2+}, pH=7.6), illuminated with 7 mW mm^{-2} 532 nm DPSS laser, and the holding potential was 0 mV. n≥6, data were presented as mean + SD. C. I–V (current–voltage) curve of CsR-2T-YE with or without LR, under same condition as in B, except the light intensity is 10 mW mm^{-2}. n = 6, error bars = mean ± SD. D. Comparison of the response upon green-light illumination of ApOps2-eYFP in comparison to ApOps2 2.0 in NG108-15 cells. While the protein kinetics is unchanged, the absolute pump current is strongly increased upon improvement of plasma membrane insertion in the 2.0 construct.

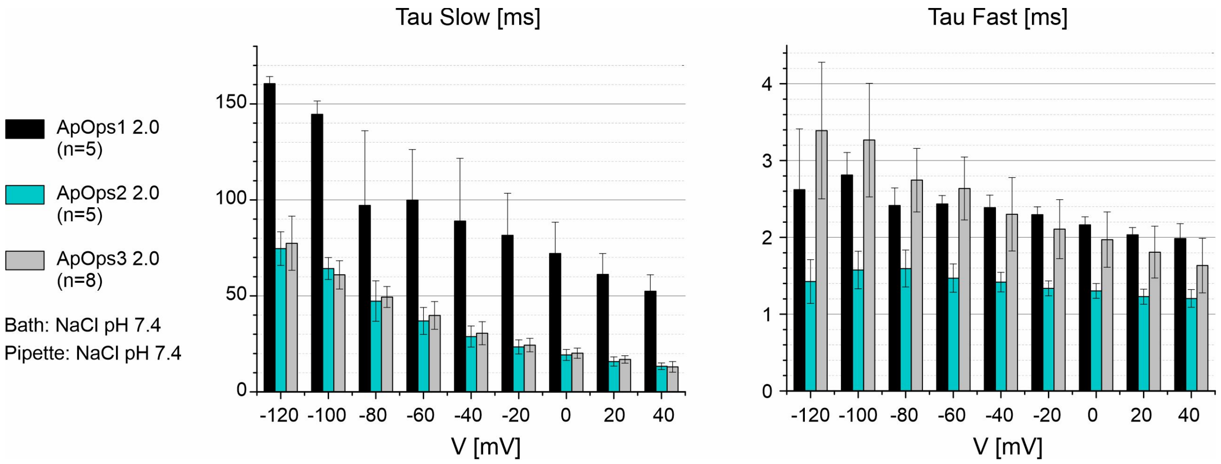

Supplementary Figure 4. Analysis of the current decay after green-light illumination. A biexponential fit is required to describe the closing kinetics observed in all three *Aureobasidium* rhodopsins. The fast component (right panel) only exhibits a low voltage dependency. ApOps1 shows the fastest time constant. The slow time constant (left) is similar for ApOps2 and ApOps3, but larger for ApOps1.

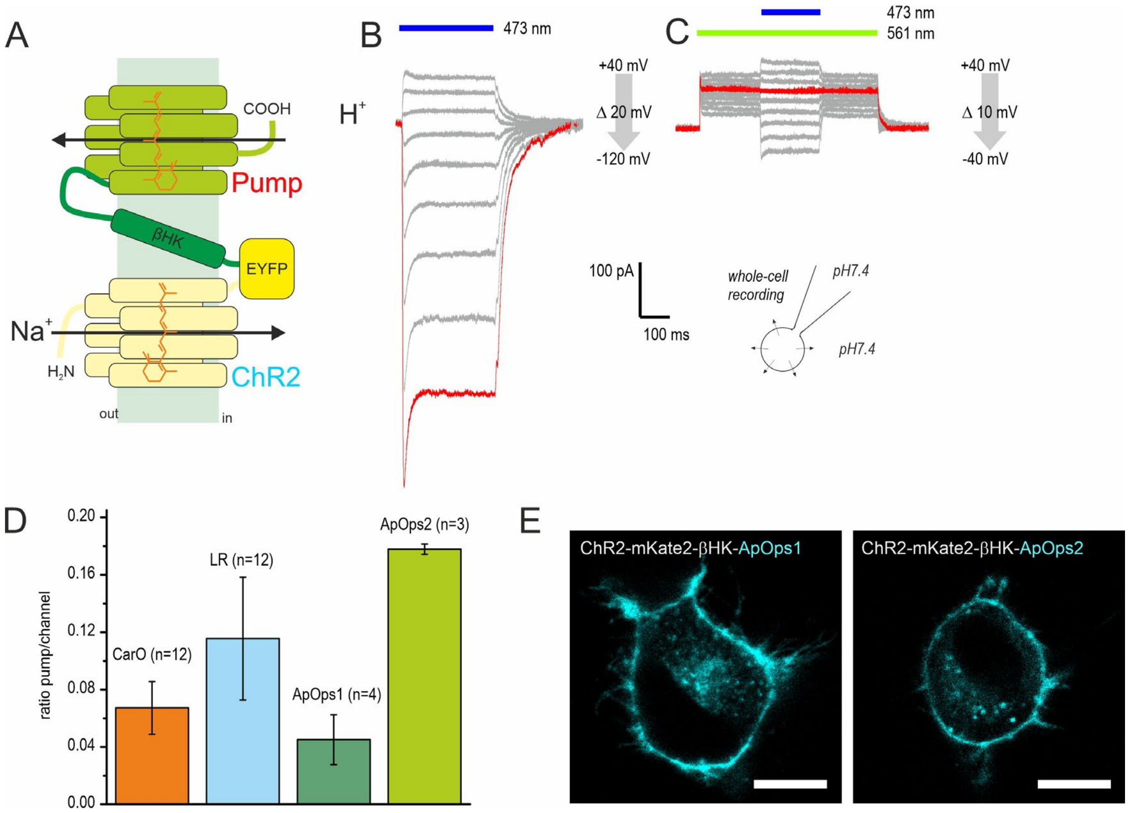

Supplementary Figure 5. Comparison of pump activity of ApOpsins with LR and CarO. **A.** Stoichometric expression of the blue light-gated channelrhodopsin-2 together with a green light-driven proton pump in a tandem cassette. **B,C.** Channel current of ChR2 at -120 mV at 473nm illumination (B) was used as a reference to compare the pump activity of the respective fungal rhodopsin upon illumination with green light (561 nm) at 0 mV (C). At 0 mV the contribution of ChR2 to the pump current is marginal, allowing for direct comparison of the pump activity of the respective fungal rhodopsin. **D.** Ratio of pump current at 0 mV to ChR2-current at -120mV. ApOps2 show higher relative pump currents than LR, which exhibits almost double intensity of CarO and ApOps1. **E.** CLSM images showing the expression of the ApOps1/2-tandem constructs in NG108-15 cells. Scale bar, 10 µm.

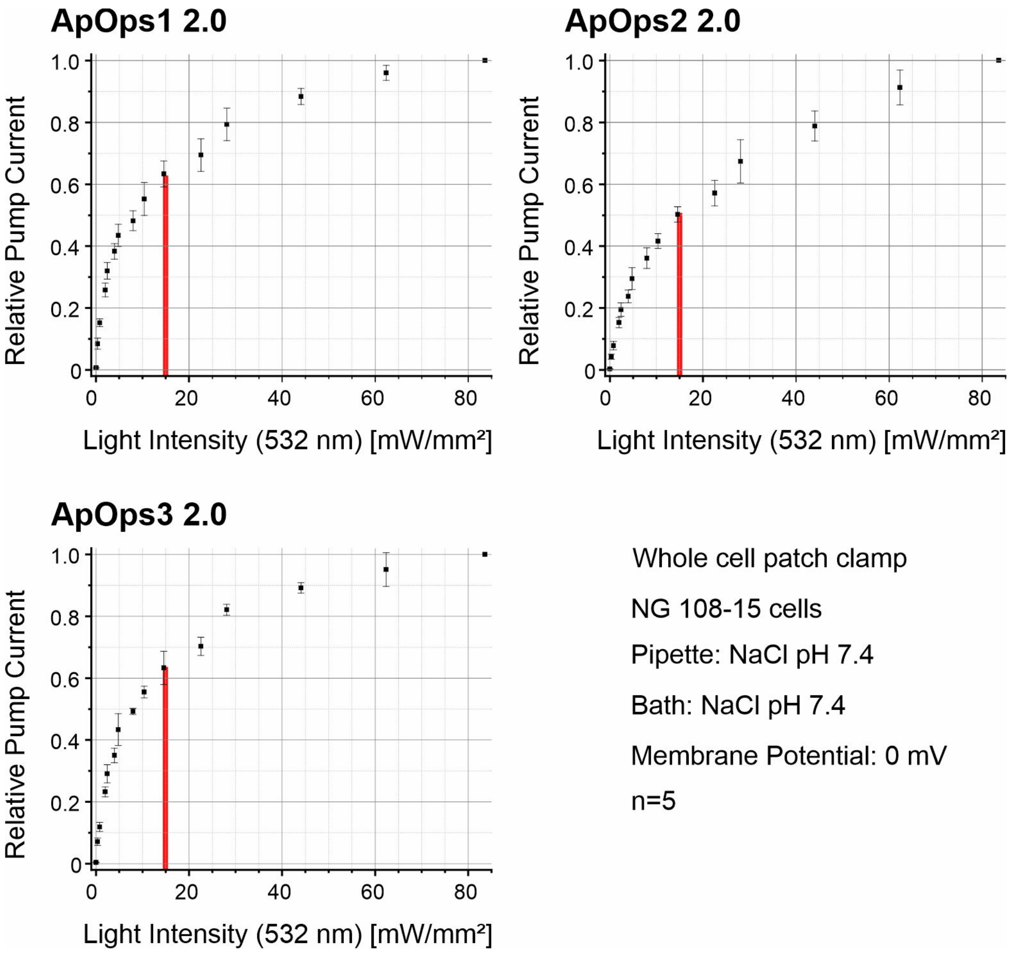

Supplementary Figure 6. Dependence of the pump activity on the laser power used for illumination. The relative pump current is given as the percentage of the value observed at 84 mW mm^{-2}. Mean value and standard deviation of 5 independent measurements. All three rhodopsins are not saturating at 15 mW mm^{-2} (indicated by the red bar).

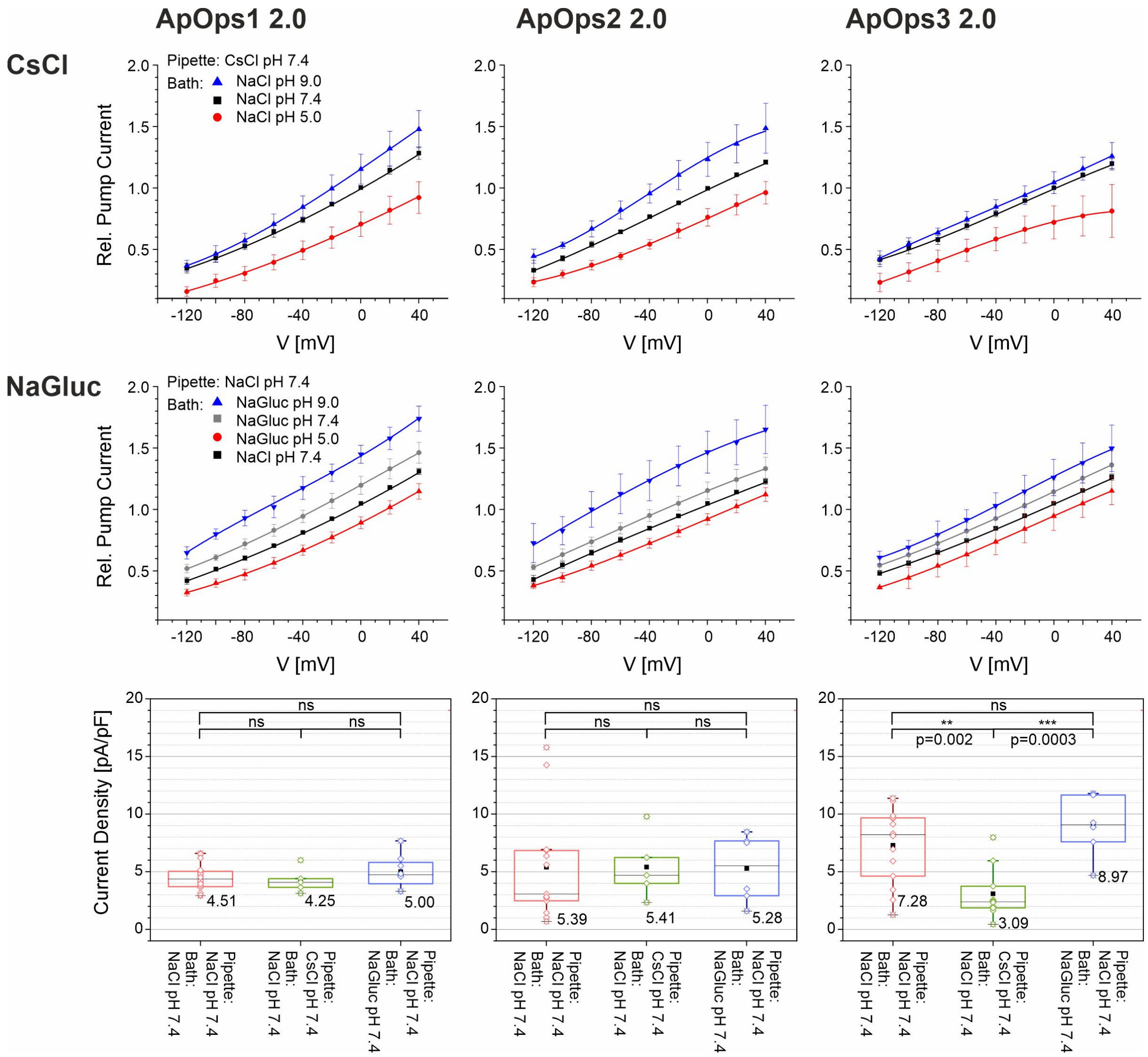

Supplementary Figure 7. Electrophysiological analysis of the pump activity after replacement of intracellular sodium by cesium and extracellular chloride by gluconate. Top and middle: Current–voltage relation of ApOps1 2.0, ApOps2 2.0 and ApOps3 2.0 in a range of +40 to –120 mV after excitation with a 532 nm laser in different intra- and extracellular solutions as indicated. Shown is relative pump activity (mean + standard deviation of n=5 (ApOps1 2.0), n=7 (ApOps2 2.0), and n=8 (ApOps3 2.0) measurements normalized to the value obtained in bath solution NaCl pH 7.4 at 0 mV clamp voltage. Bottom, current density of ApOps1-3 after replacement of sodium or chloride as indicated. After replacement of NaCl with intracellular CsCl a very significant decrease in current density is observed for ApOps3 2.0.

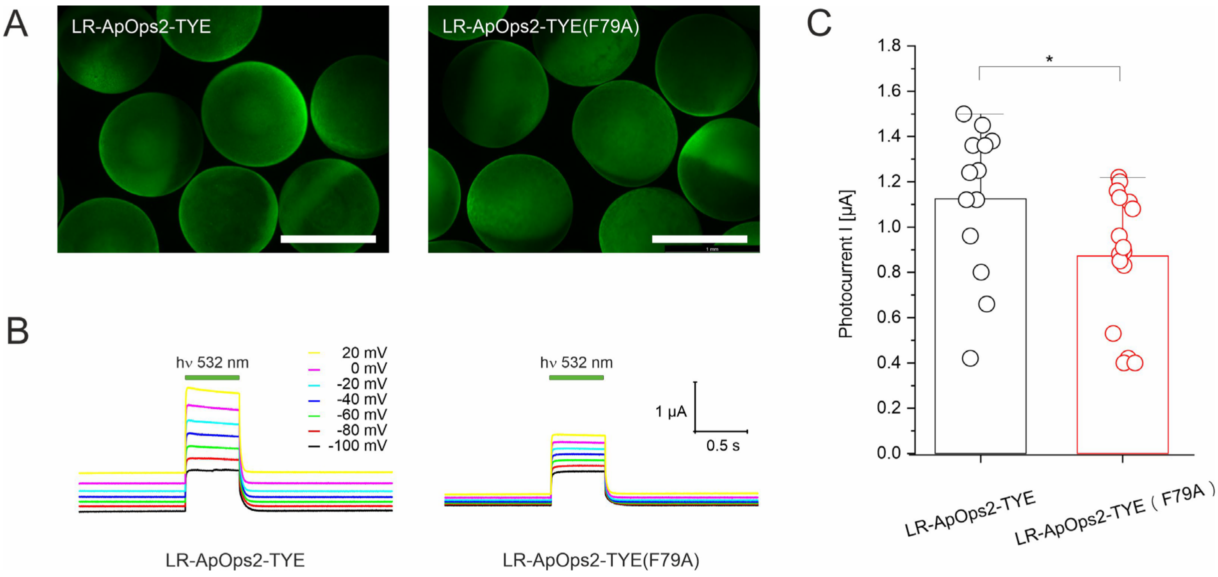

Supplementary Figure S8 Characterization of F79A mutant of LR-ApOps2-TYE. Comparison of the expression (A), photocurrent trace (B) and current magnitude (C) of LR-ApOps2-TYE and its F79A mutant in *Xenopus* oocytes. The white scale bar in (A) indicates 1 mm. The data of (B) and (C) were obtained from TEVC measurement. The experiment was performed in Ori buffer (2mM Ba^{2+}, pH = 7.6), illuminated with 10 mW mm^{-2} 532 nm DPSS laser. For (C), n = 13-14, error bars = mean＋SD, holding at 0 mV.

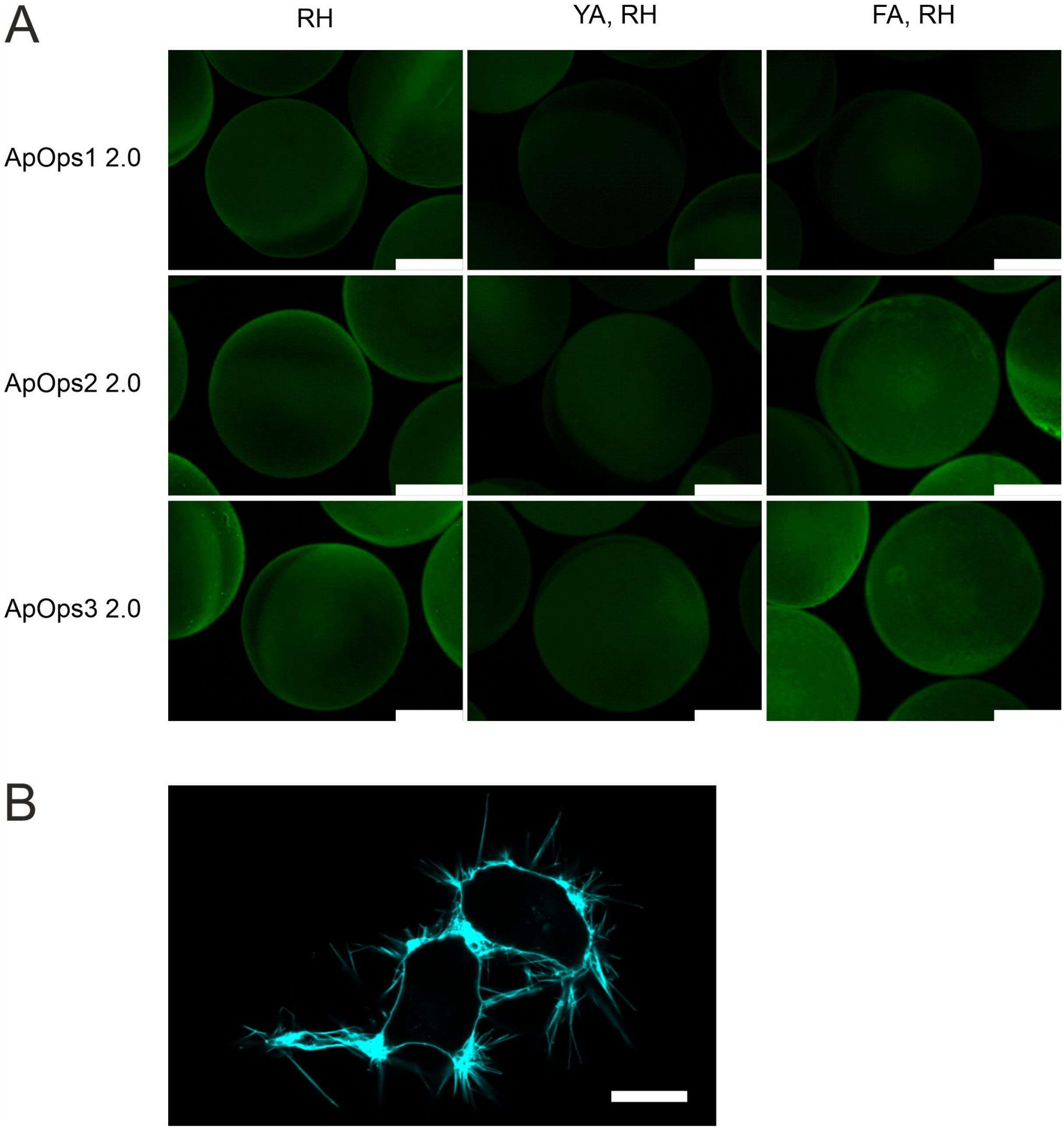

Supplementary Figure 9. Channel mutants of ApOps2 2.0. A. Expression of different proton channel mutants in *Xenopus* oocytes. Scale bar, 0.5 mm. B. CLSM image of ApOps2 2.0 F79A/R112H heterologously expressed in NG108-15 cells. The membrane trafficking of the mutants was not affected by site directed mutagenesis. Scale bar, 10 µm.
